# Differential patterns of histone acetylation in inflammatory bowel diseases

**DOI:** 10.1186/1476-9255-8-1

**Published:** 2011-01-27

**Authors:** Loukia G Tsaprouni, Kazuhiro Ito, Jonathan J Powell, Ian M Adcock, Neville Punchard

**Affiliations:** 1 Airways Disease Section, National Heart & Lung Institute, Imperial College London, Dovehouse Street, London, SW3 6LY, UK; 2 School of Health and Biosciences, University of East London, Stratford Campus, Romford Road, London, E15 4LZ, UK; 3 Gastroeintestinal Laboratory, Rayne Institute, St. Thomas Hospital, London, SE1 7EH, UK

## Abstract

Post-translational modifications of histones, particularly acetylation, are associated with the regulation of inflammatory gene expression. We used two animal models of inflammation of the bowel and biopsy samples from patients with Crohn's disease (CD) to study the expression of acetylated histones (H) 3 and 4 in inflamed mucosa. Acetylation of histone H4 was significantly elevated in the inflamed mucosa in the trinitrobenzene sulfonic acid model of colitis particularly on lysine residues (K) 8 and 12 in contrast to non-inflamed tissue. In addition, acetylated H4 was localised to inflamed tissue and to Peyer's patches (PP) in dextran sulfate sodium (DSS)-treated rat models. Within the PP, H3 acetylation was detected in the mantle zone whereas H4 acetylation was seen in both the periphery and the germinal centre. Finally, acetylation of H4 was significantly upregulated in inflamed biopsies and PP from patients with CD. Enhanced acetylation of H4K5 and K16 was seen in the PP. These results demonstrate that histone acetylation is associated with inflammation and may provide a novel therapeutic target for mucosal inflammation.

## Introduction

The cause of inflammatory bowel disease (IBD) remains unknown. The main forms of IBD are Crohn's disease and Ulcerative colitis. The main difference between Crohn's disease and UC is the *location *and *nature *of the inflammatory changes. Crohn's can affect any part of the gastrointestinal tract, from mouth to anus *(skip lesions)*, although a majority of the cases start in the terminal ileum. Ulcerative colitis, in contrast, is restricted to the colon and the rectum [1]. It has been proposed that epithelial abnormalities are the central defect, and that they underlie the development of mucosal inflammation and its chronicity [2]. In some patients IBD can be effectively treated by enemas containing short chain fatty acids (SCFA) such as butyrate, propionate, and acetate [3] in combination with steroid treatment. The molecular mechanisms that lead to this response have not been well characterized.

Several rodent models of chronic intestinal inflammation share immunopathologic features with human IBD. The two most widely used models of experimental colitis are, the 2,4,-trinitrobenzene sulfonic acid (TNBS) model of intestinal inflammation and the dextran sodium sulphate (DSS)-induced colitis model. DSS-induced colitis resembles ulcerative colitis with regard to its pathologic features. The TNBS induced colitis is an experimental model of intestinal inflammation that most closely resembles the histologic features of Crohn's disease [4,5]. It has recently been reported that distinctive disease-specific cytokine profiles were identified with significant correlations to disease activity and duration of disease in the two models. TNBS colitis exhibits a heightened Th1-Th17 response (increased IL-12 and IL-17) as the disease becomes chronic. In contrast, DSS colitis switches from a Th1-Th17-mediated acute inflammation to a predominant Th2-mediated inflammatory response in the chronic state [6,7].

Two recent articles clearly show that the transcription factor NF-B signalling in intestinal epithelial cells plays a crucial role in controlling inflammatory responses and fighting infection in the gut [8,9]. In addition, p65 antisense oligonucleotides [10] and NF-κB inhibitors [11,12] block inflammation in DSS induced colitis. NF-κB enhances inflammatory gene expression by recruiting transcriptional co-activator proteins that have intrinsic histone acetyltransferase activity [13]. Remodelling of chromatin within the nucleus, controlled by the degree of acetylation/deacetylation of histone residues on the histone core around which DNA is coiled, is important in allowing access for transcription factor DNA binding and hence gene transcription. Nuclear histone acetylation is a reversible process and is regulated by a group of acetyltransferases (HATs) which promote acetylation, and deacetylases (HDACs) which promote deacetylation. HDAC inhibitors such as butyrate and TSA can function by triggering the NF-κB response, resulting in enhanced expression of NF-κB-dependent inflammatory genes [14,15]. Non-selective HDAC inhibitors can ameliorate experimental colitis in mice by suppressing cytokine production, inducing apoptosis and histone acetylation [16] possibly relating to inflammatory cell survival although their precise mechanism of action is unclear [17,18]. The effect of the HDAC inhibitors could also be due to the large number of non-histone targets [18] including transcription factors such as NF-κB, cytoskeletal proteins and cell cycle regulators thereby affecting not only inflammatory gene expression but cell proliferation and survival [19,20].

NF-κB-induced lysine residue-specific histone acetylation (K8 and K12) has been associated with up-regulation of inflammatory genes in some cells whereas gene induction by nuclear receptors such as the glucocorticoid receptor is linked to acetylation of different lysine residues [21]. In more recent studies, reduced dexamethasone-induced transactivation in CD8^+ ^T cells compared to CD4^+ ^T cells was shown and was related to attenuated H4 lysine 5 acetylation in response to dexamethasone [22]. The importance of specific lysine histone acetylation is also stressed by Fraga and colleagues who showed that global loss of acetylation lysine16 and trimethylation of lysine 20 of histone 4 is a common hallmark of human tumour cells [23]. Here, we investigate the pattern of histone 4 acetylation and its localization in two *in vivo *models of inflammation and in patients with Crohn's disease.

### Experimental Procedures

#### Animal tissue samples

Two models of experimental colitis were chosen in order to depict different pathologic features associated with Crohn's disease and Ulcerative colitis and to possibly compare different patterns of histone acetylation with different pathologic features. The 2,4,-trinitrobenzene sulfonic acid (TNBS) model of intestinal inflammation, based on that of Morris *et al.*, was used [24]. Tissue was kindly provided by UCB, Slough, UK. The studies were performed in accordance with the UK Home office procedures. Eighteen male Sprague-Dawley rats (median weight of 337.5 g) and eighteen male Lewis rats (media weight 205 g) (Charles River, UK) were used. All rats were allowed free access to standard pellet chow and water *ad libitum*. They were randomly assigned into two groups. The first group was treated intra-rectally with 30 mg of TNBS in 30% w/v ethanol, on day zero. The second, Sham operated (control), was treated with 30% ethanol alone. The animals were sacrificed on day seven and tissue was resected from two separate areas of the large intestine- two centimetres distal to the caecum (proximal colon) and three centimetres proximal to the anus (distal colon). Within the TNBS treated group these two areas constituted the inflamed (distal) and non-inflamed (proximal) regions of the colon. For the dextran sodium sulphate (DSS)-induced colitis model, colonic inflammation was induced to Spraque-Dawley and Lewis rats by administration of 5% DSS (molecular mass, 40 kDa, ICN Biomedical, Aurora, OH) in filter purified (Millipore Bedford, MA) drinking water for 8 days as previously described [25].

#### Human tissue samples

Human tissue was collected during routine surgery, or routine endoscopy procedures at St. Thomas' hospital with appropriate ethical approval. Biopsies were collected from 12 patients aged between 18-57 yrs with Crohn's disease from macroscopically inflamed or non-inflamed regions of the large and small intestine or were isolated Peyer's patches and were grouped to inflamed and non-inflamed based on macroscopic examination. The patients were undergoing treatment with sulfasalazine and/or antibiotics (ampicillin, tetracycline). None of the patients were smokers. Inflammation was graded using a previously validated scoring system according to the cellularity of the lamina propria and the severity of changes in the enterocytes and crypts. In this system, grade 0 represents no inflammation, termed 'non-inflamed', and grade 3, represents severely inflamed biopsies. Any samples from macroscopically non-involved areas that showed evidence of microscopic inflammation were excluded from analysis. Samples of bowel were also taken from 11 patients undergoing intestinal resection for carcinoma of the colon, to serve as non-inflamed controls. Biopsies were collected at least 4 cm from macroscopic disease [26]. All samples were snap frozen in liquid nitrogen immediately after excision. Tissue was subsequently maintained in a frozen state at -80°C until use.

### Preparation of tissue sections

For microscopic analysis, the biopsies were fixed in 4% (w/v) paraformaldehyde/PBS for 3 h at 4°C, cryoprotected in sterile 4% (w/v) sucrose/PBS at 4°C overnight, mounted in OCT mountant (BDH, Atherstone, UK) on labeled cork discs and frozen in liquid nitrogen-cooled isopentane. Tissue samples were stored at -80°C. The tissues were sectioned (8 μm), mounted and the slides allowed to air-dry, covered in foil and stored at -20°C.

### Direct Histone Extraction

Histones were extracted from nuclei, as previously described by Ito *et al.*, [27]. In brief, tissue was frozen in liquid nitrogen and minced in a pestle and mortar. The homogenate was collected in 100 μl PBS, microcentrifuged for 5 min and then extracted with ice-cold lysis buffer (10 mM Tris-HCL, 50 mM sodium bisulfite, 1% Triton X-100, 10 mM MgCl_2_, 8.6% sucrose, complete protease inhibitor cocktail [Boehringer-Mannheim, Lewes, UK]) for 20 min at 4°C. The pellet was washed in buffer three times (centrifuged at 8.000 rpm for 5 min) and the nuclear pellet was washed in nuclear wash buffer (10 mM Tris-HCL, 13 mM EDTA) and resuspended in 50 μl of 0.2 N HCL and 0.4 N H_2_SO_4 _in distilled water. The nuclei were extracted overnight at 4°C and the residue was microcentrifuged for 10 min. The supernatant was mixed with 1 ml ice-cold acetone and incubated overnight at -20°C. The sample was centrifuged for 10 min, washed with acetone, dried and diluted in distilled water. Protein concentrations were determined using a Bradford method based protein assay kit (Bio-Rad, Hemel Hempstead, UK).

### Immunoblotting

Isolated histones were measured by sodium dodecyl sulfate-polyacrilamide gel electrophoresis (SDS-PAGE) [28]. Proteins were size fractionated by SDS-PAGE and transferred to Hybond-ECL membranes. Immunoreactive bands were detected by ECL. 30-50 μg of protein were loaded per lane. The following antibodies were used at a 1:1000 dilution: (pan-acetylated H4, pan-acetylated H3, H4-K5, H4-K8, H4-K12 and H4-K16 (all from Serotec, Oxford, UK). β-actin was used as internal control at a dilution of 1:10000 (Abcam, Cambridge, UK). The secondary antibody used was 1:4000 rabbit anti-goat or goat anti-rabbit antibody (Dako) linked to horseradish peroxidase. Bands were visualized by enhanced chemiluminescence (ECL) as recommended by the manufacturer (Amersham Pharmacia Biotech, Little Chalfont, UK) and quantified using a densitometer with Grab-It and GelWorks software (UVP, Cambridge, UK). The individual band optical density values for each lane were expressed as the ratio with the corresponding ß-actin optical density value of the same lane.

### Immunohistochemistry

The slides were fixed for 10 min in chilled acetone and allowed to air dry for a further 10 mins. They were then incubated for 1 hr in Quench Endogenous Peroxidase (3% H_2_O_2 _in PBS containing 0.02% Sodium Azide). Subsequently, they were washed 3 × 5 mins in PBS and pre-blocked with 5% normal swine serum (Serotec, Oxford, UK) for 20 mins. The slides were incubated with the primary antibody (pan-acetylated H4, pan-acetylated H3, H4-K5, H4-K8, H4-K12 and H4-K16 [Serotec, Oxford, UK]) diluted in PBS, at 1/100 dilution, for 2 hr. They were then washed twice for 5 mins in PBS and incubated with biotinylated swine anti-rabbit immunoglobulin G (IgG, DACO), 1/200 dilution, for 45 min. Slides were washed in PBS, distilled water and counterstained in 20% Harris haematoxylin for 10 sec. Finally, they were air-dried and mounted in DPX. Micrographs were captured using a light microscope (Leitz Biomed, Leica, Cambridge) linked to a computerized image system (Quantimet 500, Software Qwin V0200B, Leica) [28,29].

### Statistics

Results are expressed as mean ± standard error of the mean (SE). A multiple comparison was made between the mean of the control and the means from each individual group by Dunnett's test by using SAS/STAT software (SAS Institute Inc., Cary, N.C.). We performed all statistical testing by using a two-sided 5% level of significance.

## Results

### Macroscopical characterisation of the intestine in a rat TNBS model of colitis

**Figure 1:**
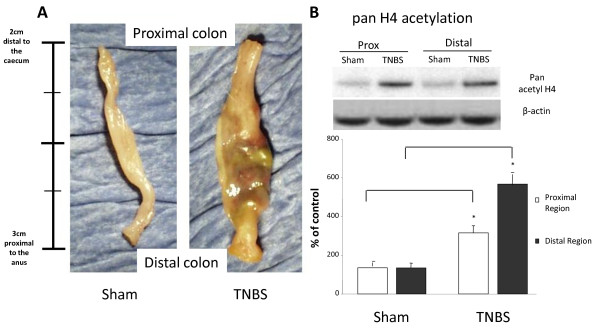
**Acetylation on histone 4 in the trinitrobenzene sulfonic acid (TNBS) rat model of inflammation**. **A**: Sham (saline treated) operated and TNBS treated rat large intestine. Rats were Sham or TNBS treated for 7 days before sacrifice. Well-advanced inflammation is apparent in the colon of the TNBS rat model. **B**: Pan acetylation on histone 4 (H4). The Sham model was saline-treated and therefore less inflamed (control). Results were obtained by Western blotting. The ratio of the density of histone H4 bands over β-actin control bands was calculated. In order to evaluate changes in density from different Western blotting experiments control densitometry was denoted as 100% and differences were accounted as increase percentage of the control. Representative examples of bands obtained are also illustrated. Columns represent the densitometric evaluation of three independent experiments (mean ± SEM). (*p < 0.05 vs Sham proximal or Sham distal respectively).

TNBS induced significant inflammation within the proximal and distal regions of the colon although the extent of inflammation was greater in the distal region (Figure 1A).

### Histone acetylation in inflamed and non-inflamed regions of the colon in the rat TNBS model of colitis

TNBS induced a significant increase in pan histone 4 acetylation in the distal (592 ± 54% vs 135 ± 24 Sham operated animals, p < 0.05) and the proximal regions of the colon (315 ± 39% vs 125 ± 19% sham operated animals, p < 0.05) with the inflamed distal region showing a greater increase (Figure 1B).

**Figure 2:**
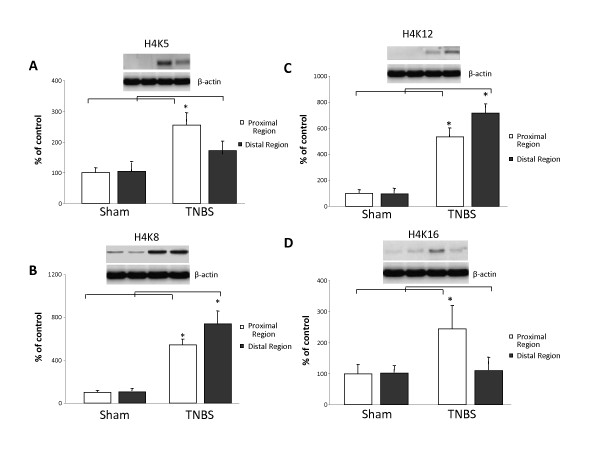
**Acetylation on histone 4 (H4) specific lysine residues 5 (K5) (A), 8 (K8) (B), 12 (K12) (C) and 16 (K16) (D) in a Sham (control) and trinitrobenzene sulfonic acid (TNBS) rat model of colitis**. Results were obtained by Western blotting. In order to evaluate changes in density from different western blotting experiments control densitometry was denoted as 100% and differences were accounted as increase percentage of the control. Representative examples of bands obtained are also illustrated. Columns represent the densitometric evaluation of three independent experiments (mean ± SEM). (*p < 0.05 vs Sham proximal or Sham distal respectively).

Acetylation of lysine (K) residues 8 and 12 were significantly increased in both the inflamed distal (K8: 818 ± 111 vs 138 ± 19%; K12: 741 ± 64 vs 121 ± 34%, both p < 0.05) and less-inflamed proximal (K8: 546 ± 50 vs 100 ± 21%; K12: 533 ± 69 vs 100 ± 26%, both p < 0.05) regions following TNBS treatment (Figure 2). However, the effect was significantly greater in the inflamed tissue than in the less-inflamed tissue for both K8 (818 ± 111 vs 546 ± 50%, p < 0.05) and K12 (741 ± 64 vs 533 ± 69%, p < 0.05).

In contrast, there was no significant induction of K5 or K16 induction by TNBS in the inflamed distal region (Figure 2). Moreover, K5 (255 ± 39 vs 100 ± 15% Sham operated animals, p < 0.05) and K16 (300 ± 63 vs 100 ± 29% Sham operated animals, p < 0.05) acetylation was enhanced in the non-inflamed proximal region.

### Localisation of acetylated histones 4 and 3 in DSS-treated animal models

**Figure 3:**
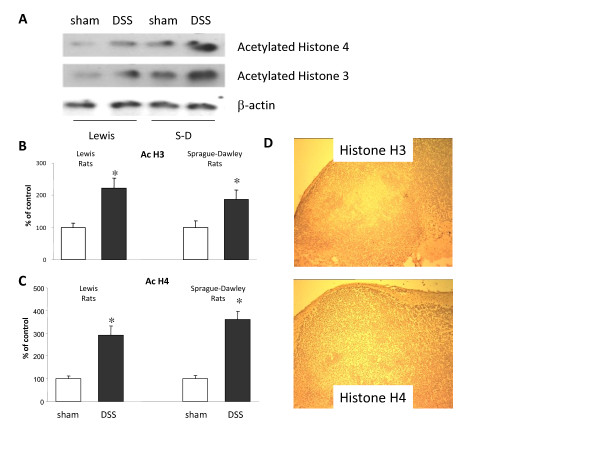
**Acetylation on histones 3 (H3) and 4 (H4) in Lewis and Sprague-Dawley dextran sulfate sodium (5% DSS) treated rats. Tissue samples were obtained from the sigmoid colon of the animals**. **A**: Representative bands of H4 and H3 acetylation as obtained by Western blotting. β-actin levels were measured to ensure equal protein loading. The results are representative of three independent experiments. **B, C**: Graphical analysis of data Lanes represent: (1) non-DSS treated Lewis rats (control), (2) DSS-treated Lewis rats, (3) non-DSS treated Sprague-Dawley rats (control) (4) DSS-treated Sprague-Dawley rats. Columns represent the mean ± SEM of three independent experiments (*p < 0.05). **D**: Histone 3 (H3) and histone 4 (H4) localisation in Peyer's patches of dextran sulfate sodium (DSS) treated Lewis rats. H3 is acetylated mainly in the mantle zone and H4 is acetylated throughout the surface of Peyer's patches to both mantle zone and germinal centre cells. In Peyer's patches of untreated animals no acetylation on either histone 3 or 4 was apparent. Micrographs are representative of two individual experiments for each strain. Isotype controls show no staining.

Acetylation of both histones 4 and 3 was evident in non-DSS treated rats but this was enhanced in all inflamed areas, regardless of distinct positions in the colon, of both for Lewis rats (H4: 222 ± 31 DSS treated vs 100 ± 31% non-DSS treated animals, p < 0.05; H3 292 ± 40 DSS treated vs 100 ± 13% non-DSS treated animals, p < 0.05) and Spraque-Dawley rats (H4: 187 ± 30 DSS treated vs 100 ± 21% non-DSS treated animals, p < 0.05; H3 361 ± 36 DSS treated vs 100 ± 15% non-DSS treated animals, p < 0.05) (Figure 3). Similar results were obtained from Sprague-Dawley DSS-treated cells.

### Localisation of acetylated histones 4 and 3 in Peyer's patches

We also investigated whether DSS-treatment would have an effect on histone acetylation in the Peyer's patches found in the small intestine. Acetylated histones are indicated by the brown colour in the micrographs. Pan acetylated H3 was situated in the mantle zone of Peyer's patches in DSS-treated Lewis and Sprague-Dawley rats in contrast to the more uniformed staining for acetylated histone 4 throughout the surface of Peyer's patches (Figure 3D).

### Specificity of histone 4 lysine acetylation in Peyer's patches after DSS treatment

**Figure 4:**
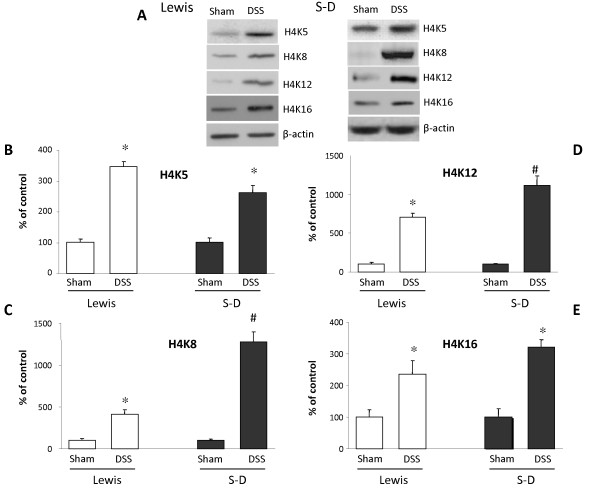
**Acetylation on histone 4 (H4) specific lysine residues 5 (K5), 8 (K8), 12 (K12) and 16 (K16) in Lewis and Sprague-Dawley dextran sulfate sodium (5% DSS)**. **A**: Representative bands of H4K5, K8, K12 and K16 acetylation. Lanes for Lewis rats represent: non-DSS treated (control) and DSS-treated. Likewise representative bands are illustrated for the Sprague-Dawley rats. Graphical representation of Western blotting data. H4 acetylation of K5 **(B)**, K8 **(C)**, K12 **(D) **and K16 **(E)**. Columns represent the mean ± SEM (bar) of three independent experiments.

DSS induced acetylation of histone 4 lysines K5, K8, K12 and K16 in both rat strains (Figure 4). However, a greater induction was seen on K8 in both Lewis (414 ± 51 DSS treated vs 100 ± 23% non-DSS treated animals) and Sprague-Dawley rats (1275 ± 123 DSS treated vs 100 ± 18% non-DSS treated animals). Similar results were seen with K12 in both Lewis (703 ± 64 DSS treated vs 100 ± 14% non-DSS treated animals) and Sprague-Dawley rats (1117 ± 113 DSS treated vs 100 ± 27% non-DSS treated animals). K5 acetylation in Lewis rats (346 ± 17 DSS treated vs 100 ± 12% non-DSS treated animals) and Sprague-Dawley rats (263 ± 22 DSS treated vs 100 ± 16% non-DSS treated animals) was also induced albeit to a lesser extent. Our findings were similar for K16 acetylation in both Lewis (235 ± 43 DSS treated vs 100 ± 22% non-DSS treated animals) and Sprague-Dawley rats (321 ± 24 DSS treated vs 100 ± 26% non-DSS treated animals).

### Histone acetylation in Crohn's disease

**Figure 5:**
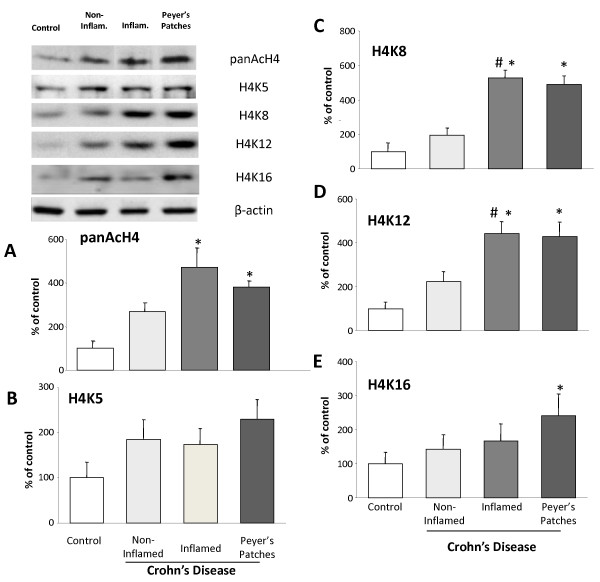
**Acetylation on histone 4 (H4) and H4 lysine residues in Crohn's disease**. Columns represent the mean ± SEM of three independent experiments. Four biopsies were pooled to obtain sufficient protein for one experiment (50 μg of protein) (*p < 0.05 vs control). Pan acetylation on H4 in Crohn's disease **(A)**. Acetylation on histone 4 (H4) specific lysine residues 5 (K5) **(B)**, K8 **(C)**, K12 **(D)**, and 16 **(E)**, in non-inflamed, inflamed tissue and Peyer's patches of Crohn's disease patients. Results were obtained by Western blotting. Columns represent the mean ± SEM of three independent experiments. (*p < 0.05 vs control, #p < 0.005 vs non-inflamed CD). Representative images of the bands obtained are illustrated.

Acetylation on H4 was slightly induced in the non-inflamed ileum of Crohn's disease patients. In contrast, H4 acetylation was significantly elevated in the inflamed regions (472 ± 88 vs 100 ± 34% control, p < 0.05) (Figure 5A). Peyer's patches from Crohn's disease patients also showed a significant increase in pan H4 acetylation (382 ± 29%) compared to the control non-inflamed tissue (100 ± 34%, p < 0.05) (Figure 5A). Levels of acetylated K5 were not significantly upregulated compared to control (Figure 5). More specifically, K8 acetylation was significantly induced compared to control samples in the inflamed regions (527 ± 44% vs 100 ± 25% control tissue, p < 0.05) and the non-inflamed CD samples (527 ± 44% vs 195 ± 42% non-inflamed CD, p < 0.05). In Peyer's patches from CD patients, K8 was significantly upregulated compared to control (488 ± 52% vs 100 ± 25% control tissue, p < 0.05) (Figure 5).

Enhanced acetylation on K12 was detected in inflamed regions of CD compared to control (442 ± 54% vs 100 ± 29% control tissue, p < 0.05) and non-inflamed CD tissue (442 ± 54% vs 223 ± 38% non-inflamed IBD tissue, p < 0.05). Similarly, enhanced acetylation on K12 was detected in Peyer's patches compared to control (429 ± 65% vs 100 ± 29% control tissue, p < 0.05). Acetylation on lysine 12 was not significantly increased in non-inflamed tissue compared to control. No changes in lysine 16 acetylation were observed in either inflamed or non-inflamed tissue from Crohn's disease patients. In the Peyer's patches, however, a significant elevation of acetylation on K16 was observed (Figure 5).

## Discussion

Our results show that acetylation of histone H4 was significantly elevated in the inflamed mucosa in the TNBS model of colitis particularly on lysine residues (K) 8 and 12 in contrast to non-inflamed tissue. In addition, acetylated H4 was localised to inflamed tissue and to PP in DSS-treated rat models. Within the PP, H3 acetylation was detected in the mantle zone whereas H4 acetylation was seen in both the periphery and the germinal centre. Finally, acetylation of H4 was significantly increased in inflamed biopsies and PP from patients with CD. Enhanced acetylation of H4K5 and K16 was seen in the PP. Acetylation of K5 and K16 was localized to the mantle zone whereas acetylation of K8 and K12 was localized to both the mantle zone and the germinal center (data not shown).The diversity of IBD and the difficulty in successfully distinguishing between Ulcerative colitis and Crohn's disease underlined the criteria for employing two different animal models for studying histone acetylation (TNBS and DSS) associated with Crohn's disease and Ulcerative colitis respectively [30].

Although in many cases it is not clear whether cytokines are the cause or the result of the underlying disease process there is little question that their presence can have profound effects upon gut epithelial cell function and that pro-inflammatory cytokines are key factors in the pathogenesis of Crohn's disease (CD). Activation of nuclear factor kappa B (NF-κB), which is involved in pro-inflammatory cytokine gene transcription, is increased in the intestinal mucosa of CD patients [31]. Modulation of histone acetylation is involved in transcriptional regulation, associated with the NF-κB pathway [32-34]. Importantly, either a lack or an excess of NF-κB can lead to IBD. As enhanced intestinal epithelial permeability may cause IBD by itself, NF-κB deficiency could underline epithelial barrier function directly by deregulating the expression of proteins involved in cellular adhesion. Alternatively, NF-κB failure could break the barrier indirectly by compromising the survival of epithelial cells [35]. This might explain the complex molecular mode of action of butyrate in IBD, where for example reports show that butyrate inhibits NF-κB activation and increases IκBβ levels in vitro in intestinal epithelial cell lines [36]. In gain of function mutations in the Nod2 gene, there is an induction of TH1 and IL-17 secreting T helper response that promotes tissue damage and Crohn's disease [37]. On the other hand, loss-of-function mutations compromise NF-κB activation and TH1 driven colitis [35].

A number of articles demonstrate that acetylation of histone H4 plays a primary role in the structural changes that mediate enhanced binding of transcription factors to their recognition sites within nucleosomes [38]. In primary airway smooth muscle cells, TNF-α induced histone 4 acetylation and this induction was attenuated by pre-treatment of cells with a glucocorticoid [39]. Finally, variations in global levels of histone marks in different grades, morphologic types, and phenotype classes of invasive breast cancer have been reported to be clinically significant [40]. The use of sodium butyrate, a histone deacetylase inhibitor, in the treatment of IBD lead to the hypothesis that in addition to its anti-proliferative action, an effect on histone acetylation could be associated with its therapeutic effects. For example, in human umbilical vein endothelial cells (HUVEC), induction of tissue-type plasminogen activator (t-PA) transcription by butyrate and Trichostatin A was preceded by histone 4 acetylation [41]. Recent evidence revealed that butyrate decreases pro-inflammatory cytokine expression via inhibition of NF-κB activation and IκBα degradation [14,18,42] while it has also been demonstrated that NF-κB induction of inflammatory gene expression is associated with histone acetylation [28,34] and indeed with p65 acetylation [43].With the importance of H4 acetylation having been studied and described in other disease models, experiments were carried out in to investigate whether acetylated histone 4 activity was altered in inflamed and non-inflamed tissue of a TNBS model of colitis. We observed differences in histone 4 acetylation levels between inflamed and non-inflamed tissue particularly with respect to K8 and K12 acetylation. This specificity towards lysine acetylation could be explained by the selective recruitment of transcriptional co-activators containing HAT activity by transcription factors such as NF-κB [44,45]. Although tempting to suggest a cause-and-effect model it is unclear whether increased inflammation leads directly to increased histone acetylation *in vivo *at specific gene promoters. Further studies will be needed to address this in IBD but preliminary evidence suggests that this may be the case for the GM-CSF promoter in alveolar macrophages from smokers [46]. Also another interesting study investigating the effect of pro-inflammatory cytokines in intestinal alkaline phosphatase (IAP) gene expression comes to further support the possible role of histone acetylation in intestinal inflammation. The authors report both histones 3 and 4 were hyperacetylated in HT-29 cells when they were stimulated with TNF-α or IL-1β concluding that both pro-inflammatory cytokines affect sodium butyrate-induced activation of the IAP gene likely via deacetylation of its promoter region [47].

Macroscopic analysis of tissue from both Lewis and Sprague-Dawley rats treated with 5% DSS revealed areas of severe inflammation. However, Peyer's patches did not show any signs of inflammation agreeing with previous results showing that the DSS model resembles ulcerative colitis with inflammation present in the descending and sigmoid colon and the rectum but is not apparent along the wall of the small intestine where Peyer's patches are situated. In the DSS model, acetylation of histones 4 and 3 was upregulated in both Lewis and Sprague-Dawley rats. Comparison of acetylated levels between histones 3 and 4 revealed that while both were acetylated, the latter reached significantly higher levels. Similarly, in Peyer's patches of the DSS model, histone 4 acetylation was greater than that of histone 3. Immunohistochemical investigation of Peyer's patches revealed a distinct pattern of histone acetylation. Acetylation on H3 was only detected in the mantle zone of Peyer's patches, whilst acetylated H4 occurred in both the periphery and the germinal centre of Peyer's patches. Therefore, it was concluded that acetylation on H3 could possibly be cell specific, whereas H4 is generally induced in all cell types present in Peyer's patches (T-cells, B-cells, dendritic cells and macrophages) although this needs to be formally assessed (possibly by counter staining). These data indicate an increase in histone acetylation during gut inflammation. In support, a number of reports show differential H3 acetylation patterns between TH1 and TH2 cells [48,49].

Acetylation of K8 and K12 is associated with the upregulation of inflammatory genes [28]. In the DSS model of colitis, H4 K8 and K12 were highly acetylated in the Sprague-Dawley rats. These findings were in agreement with previous results documented *in vitro *[50]. Interestingly, in the Lewis rats, only K12 acetylation was strongly induced. This difference could be attributed to genetic variances between the two rat strains, as discussed by other groups [51,52].

The present study was concluded by measuring H4 acetylation in Crohn's disease patient biopsies. As with the TNBS model, Peyer's patches, non-inflamed and inflamed biopsies were assessed. Levels of acetylated H4 were most prominent in the inflamed biopsies, followed by those in Peyer's patches albeit to a lesser extent. Acetylation was also detectable in the non-inflamed mucosa of Crohn's disease patients. The results for acetylation on H4 lysines in Crohn's disease were very similar to those obtained in the TNBS treated animals. K5 and K16 were only slightly acetylated in all samples, with the inflamed and non-inflamed samples presenting no significant difference in acetylation. Peyer's patches showed the highest levels of K5 and K16 acetylation. Finally, in biopsies of inflamed bowel and in Peyer's patches of Crohn's disease patients, K8 and K12 were both significantly acetylated. Acetylation on lysine residues in the non-inflamed biopsies was only slightly upregulated. The results suggested that although pan acetylation on H4 in the Peyer's patches is probably not cell specific, it is possible that acetylation of its specific lysine residues is cell type dependent. This could also explain the significant increase in K8 and K12 acetylation revealed by Western blotting. An increased Treg number in Peyer's patches indicates that they have a very important niche in the peripheral gut, where new encounters with antigens are very critical. In this respect, it seems natural that Treg are more numerous in Peyer's patches as it is in the gut that antigens to cross the intestinal barrier are to be processed and exert their effect, and thus it is an area where essential antigenic surveillance is taking place [53].

Site specific histone acetylation and deacetylation have been associated in more recent years with a number of different functions such as nucleosome assembly, heterochromatin silencing, transcription and gene repression [54]. The human chromatin assembly factor 1 (CAF-1) complex co-purifies with histone H4 modified at sites that are indicative of recent synthesis. Acetylation is observed at K5, K8 and/or K12 but not at K16 [55]. In yeast H4K16 appears to be critical for the silencing information regulator protein (Sir) binding because the interaction between full length Sir3 and an H4 peptide in vitro is abolished by acetylation of lysine 16 but not other lysines [56]. Another example of site specific lysine acetylation involves the SMRT mammalian co-repressor. SMRT preferentially binds to the unacetylated histone 4 tail and its binding is dependent on deacetylated H4K5 [57]. Finally, another example of the effect of specific lysine residue acetylation in gene function is the observation that with the coding region of ERG11, an active gene, deacetylases Hos2 and Rpd3 redundantly deacetylate all lysines in histone 4 and H4 tails except for H4K16, which is deacetylated primarily by Hos2 [58]. Precise patterns of acetylation at promoters, therefore, may be recognized by particular transcription factors because specific combinations of hypoacetylated residues at genes correlate with specific expression profiles over a variety of conditions [54].

Paradoxically, HDAC inhibitors are used in the treatment of IBD. This may reflect either an anti-proliferative effect seen with high, non-specific doses of HDAC inhibitors or an effect on the acetylation status of non-histone proteins e.g. tubulin and transcription factors such as NF-κB and GATA [20,59,60]. Recent reports, however, show that administration of an HDAC inhibitor *in vivo *increased Foxp3 gene expression, as well as the production and the suppressive function of regulatory T cells (Treg cells). It has been shown that HDAC inhibition therapy *in vivo *enhanced Treg-mediated suppression of a homeostatic proliferation and decreased IBD through Treg-dependent effects [61]. These results may, at least in part, reflect the activation of regulatory T-cells involved in active NF-κB suppression (and increased histone acetylation) of inflammation primarily induced in the Peyer's patches [62].

The results presented here are indicative of the importance of histone 4 acetylation in the expression of inflammatory genes in inflammatory diseases, such as IBD. Whether this is causal or downstream to activation of inflammation is unclear but suggests that HAT inhibitors may be useful in treatment. Deacetylase inhibitors *in vivo*, such as Belinostat (PXD101) and Phenylbutyrate, are currently used in clinical trials. However, most clinical trials have not had much success either due to the disease being stable or due to adverse effects of the drug [63]. The mechanism might be better understood when the target proteins (histone or non-histone) of these compounds are identified.

The present preliminary studies aim to provide further understanding in the role that histone acetylation plays in the regulation of inflammation. Future studies should examine the activity of specific HATs and HDACs in individual immune and resident cells types. It is, therefore, possible to speculate that further understanding of the role of histone modifications in IBD may lead to new therapeutic strategies in the treatment of IBD and explain the therapeutic utility of current treatment.

## Competing interests

The authors declare that they have no competing interests.

## Authors' contributions

LGT performed all experiments and drafted the manuscript. KI participated in the histone extraction methods. JJP provided clinical and animal samples. IMA participated in the design and coordination of the study and to manuscript writing. NP participated in the design and coordination of the study. All authors read and approved the final manuscript.
